# Effect of Transcranial Direct Current Stimulation on Functional Capacity in Schizophrenia: A Study Protocol for a Randomized Controlled Trial

**DOI:** 10.3389/fpsyt.2017.00233

**Published:** 2017-11-13

**Authors:** Zui Narita, Takuma Inagawa, Kazushi Maruo, Kazuki Sueyoshi, Tomiki Sumiyoshi

**Affiliations:** ^1^Department of Psychiatry, National Center Hospital, National Center of Neurology and Psychiatry, Tokyo, Japan; ^2^Department of Clinical Epidemiology, Translational Medical Center, National Center of Neurology and Psychiatry, Tokyo, Japan

**Keywords:** neuromodulation, daily-living skills, cognition, transcranial direct current stimulation, functional outcome, randomized controlled trial

## Abstract

Schizophrenia patients elicit a wide range of psychopathology, including psychotic symptoms, mood symptoms, and cognitive impairment. Functional capacity is defined as the ability to perform everyday living skills, which is linked to cognition and real-world functional outcome. In a previous open trial, we demonstrated that transcranial direct current stimulation (tDCS), one of the neuromodulation methods, improved cognition and functional capacity in 28 patients with schizophrenia. However, since it was a pilot study, a controlled trial is needed. Therefore, we present a study protocol for a randomized controlled trial designed to evaluate the effect of tDCS on functional capacity in patients with schizophrenia. This is a two-arm, parallel-design, randomized controlled trial, in which patients and assessors will be blinded. Patients meeting DSM-5 criteria for schizophrenia will be enrolled and randomized to receive either active or sham stimulation (with 10 sessions in five consecutive days). Functional capacity will be evaluated by the UCSD Performance-based Skills Assessment-Brief as primary outcome. Cognition, as measured by the Brief Assessment of Cognition in Schizophrenia, and psychotic symptoms, as measured by the Positive and Negative Syndrome Scale, will also be evaluated. Data will be collected at baseline, immediately after the last stimulation, and 1 and 2 months thereafter. If active stimulation elicits greater effects compared with those of sham stimulation, it may add to the efforts to improve functional outcomes by neuromodulation in patients with schizophrenia.

**Trial registration:**

UMIN000028224; https://upload.umin.ac.jp/cgi-open-bin/ctr/ctr_view.cgi?recptno=R000032305.

## Introduction

Schizophrenia patients elicit a wide range of psychopathology, including psychotic symptoms, mood symptoms, and cognitive impairment (1–3). Several domains of cognitive function such as some types of memory, executive function, verbal fluency, and attention/information processing are impaired in schizophrenia (1, 2). Functional capacity is defined as the ability to perform everyday living skills, such as financial competence and communication in controlled and observational settings (3). By contrast, social function is influenced by several factors, such as opportunities and incentives in daily life (4). These levels of outcomes (cognitive function, functional capacity, and social function) have been reported to be related to each other (5, 6).

The UCSD Performance-based Skills Assessment-Brief (UPSA-B) is a scale of functional capacity linked to cognitive functioning in schizophrenia (5, 7). Patients with the illness demonstrate significantly lower scores on this scale compared with healthy controls, a finding pertinent to some of the non-Western countries, including Japan (5, 7).

Neuromodulation is defined as alterations of neural activity with targeted delivery of a stimulus, such as electrical stimulation or chemical agents, to specific neurological sites in the body. Neuromodulation techniques range from non-invasive approaches, e.g., transcranial magnetic stimulation, to invasive (implanted) devices, e.g., spinal cord stimulation and deep brain stimulation. For instance, transcranial direct current stimulation (tDCS) is a feasible and safe method, using weak and direct electrical current to the brain through electrodes (8, 9). Typically, two electrodes are placed over the scalp, through which anodal and cathodal stimulation increases and decreases cortical excitability, respectively. With this mechanism, tDCS has been suggested to modulate corticosubcortical/corticocortical pathways (10, 11).

tDCS has been shown to improve several domains of cognitive function in healthy subjects, stroke patients, and elderly individuals (12–17). Also, some studies reported facilitative effects of tDCS on learning memory, working memory, and verbal fluency in schizophrenia (18–20). Moreover, one sham-controlled randomized study has revealed that tDCS improved performance on the MATRICS Consensus Cognitive Battery in schizophrenia (21). On the other hand, little information is available about the effect of tDCS on functional capacity, as evaluated by a specific assessment tools, such as the UPSA-B.

In a previous open trial, we demonstrated the ability of tDCS to improve functional capacity, as well as depressive symptoms, in patients with schizophrenia (22). However, since this was a pilot study with a single arm, conducting a controlled trial is desirable. Therefore, we present a study protocol for a randomized controlled trial designed to evaluate the efficacy of tDCS on functional capacity in patients with schizophrenia.

## Material and Equipment

### Study Design

**Figure 1:**
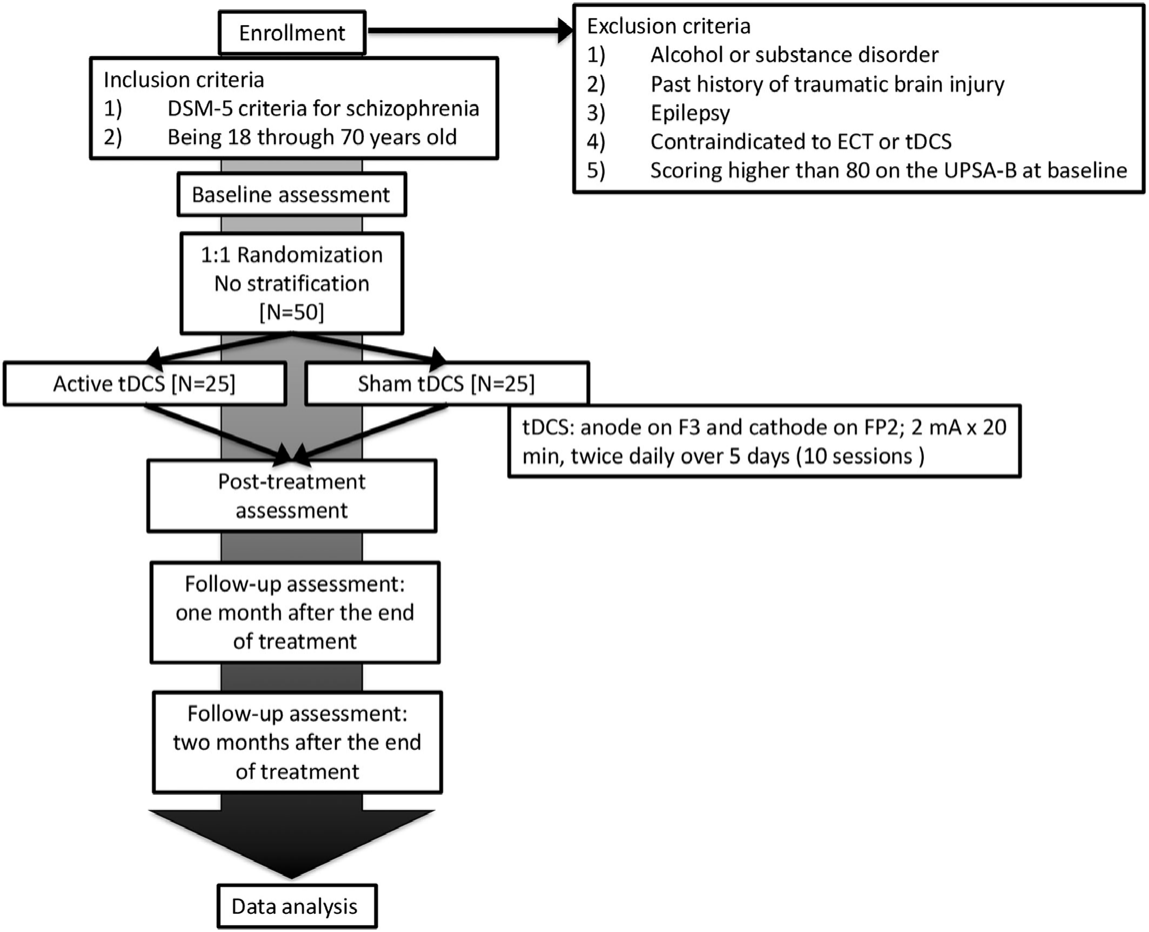
Flowchart summarizing the trial procedure. ECT, electroconvulsive therapy; tDCS, transcranial direct current stimulation.

This is a single-center trial at Nacional Center of Neurology and Psychiatry, Tokyo, Japan (Figure 1). A two-arm, parallel-design, randomized controlled trial is planned, in which patients and raters will be blinded. Participants will be randomized with 1:1 ratio to either active or sham tDCS group with computer-generated sequence in the Electronic Data Capture (EDC) system. The superiority of active tDCS to sham tDCS will be investigated. Subjects will receive 10 sessions of active/sham tDCS in five consecutive days (twice per day). A participant’s allocation will be revealed by the principle investigator after the study endpoint. Results of the trial will be communicated by the study coordinators when requested.

### Participants

Inpatients or outpatients treated at National Center Hospital, National Center of Neurology and Psychiatry will be enrolled. Subjects will be recruited by referrals from treating psychiatrists. They must provide written informed consent before starting the trial.

Subjects must meet the following inclusion criteria:

(1) DSM-5 criteria for schizophrenia
(2) Being 18 through 70 years old

Patients with any of the following conditions will be excluded from the study:

(1) Alcohol or substance disorder
(2) Past history of traumatic brain injury
(3) Past history of epilepsy
(4) Contraindicated to electroconvulsive therapy or tDCS
(5) Scoring higher than 80 on the UPSA-B at baseline

The dose of neuroleptics will not be changed during the study period. In our pilot study (22), mean and SD of change from baseline of the UPSA-B was 11.68 (9.84) in patients scoring lower than 80 at baseline. We conservatively hypothesized that 9.00, the lower limit of 50% confidence interval of mean change from baseline in the pilot study, would be the mean difference between two groups in this study. In addition, Pearson correlation coefficient between the change and baseline value of the UPSA-B was estimated as −0.340. In these conditions, with a power of 0.9 for the primary analysis, an approximate number of 24 per group were estimated. Thus, considering study dropouts, a total sample of 50 was determined to be included.

### Intervention

Direct current will be transferred by 35-cm^2^ saline-soaked sponge electrodes and delivered by Soterix Medical 1 × 1 Transcranial Direct Current Low-Intensity Stimulator Model 1300A. For each session, the tDCS montage will comprise placement of the anode over the left dorsolateral prefrontal cortex and the cathode over the contralateral supraorbital area, which corresponds to F3 and FP2 area, according to the International 10–20 electroencephalography system. We will apply 10 sessions of direct current of 2 mA for 20 min in five consecutive days (twice per day, 10:00 a.m. and 2:00 p.m.). The dose and frequency of stimulation were determined based on the pilot study (22).

For the sham group, the device will be turned off after 1 min of active stimulation. The electrode position and all the other procedures, including electrode moisture and checking the contact, will be identical to the conditions for the active-stimulation group. The display of the device will be kept outside participants’ vision field, and the device will be turned off blinded to subjects. A controlled study reports blinding integrity of tDCS and pharmacological treatment was comparable (23). The assessors and patients will be blinded to the treatment, and the contact between participants will be avoided to enhance the effect of study blinding.

Trained psychiatrists will administer tDCS. Since they will not be blinded, his or her interaction with participants will be minimized. Also, the experimenters will not participate in the assessment of outcome measures or any other aspects of the trial. To improve adherence, we will provide all consenting participants with costs of transportation and will remind and reschedule the visits of participants if necessary.

### Outcome Measures

Patients will be assessed after being informed of the objectives of the study and providing their informed consent to participate. Data will be collected following an assessment that will be implemented at baseline, immediately after the last stimulation, and 1 and 2 months thereafter (see Table 1). Baseline and follow-up evaluations will be performed by experienced psychologists blinded to group allocation.

**Table 1:** Study schedule.

	Study period							
	Enrollment	Intervention					Follow-up 1	Follow-up 2

Time point	Week 1	Week 2					Week 6	Week 10
		Day 1	Day 2	Day 3	Day 4	Day 5		
**Enrollment**								
Eligibility screen	X							
Informed consent	X							
Sociodemographic characteristics	X							
**Intervention**								
Transcranial direct current stimulation (twice/day)		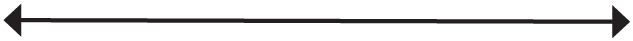						
**Assessments**								
UPSA-B	X					X	X	X
BACS	X					X	X	X
PANSS	X					X	X	X
Adverse events	X	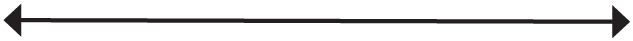					X	X

*UPSA-B, the UCSD Performance-based Skills Assessment-Brief; BACS, the Brief Assessment of Cognition in Schizophrenia; PANSS, Positive and Negative Syndrome Scale*.

#### Functional Capacity (Daily-Living Skills)

The primary outcome is functional capacity evaluated by the UPSA-B (7), which consists of Finance and Communication subscales.

#### Cognition

Cognitive function will be assessed by the Brief Assessment of Cognition in Schizophrenia (BACS), which includes tests of verbal memory (Verbal Memory Task), verbal working memory (Digit Sequencing Task), motor/speed (Token Motor Task), verbal fluency (Verbal Fluency Task), attention/information processing (Symbol Coding Task), and executive function (Tower of London Task). To provide a standard metric for combining test scores into domains and comparing performance over time, BACS scores will be converted to *z*-scores, which shows relative outcomes compared with those of healthy people (1). Alternative forms will be used for Verbal Memory Task and Tower of London Task at baseline and follow-up assessments.

#### Psychotic Symptoms

Psychotic symptoms will be evaluated by the Positive and Negative Syndrome Scale (PANSS), commonly used for the assessment of psychotic symptoms of schizophrenia (24). It consists of Positive Syndrome, Negative Syndrome, and General Psychopathology subscales.

To ensure the success of blinding, we will ask patients to guess whether the treatment was active or sham after the stimulation procedure has been completed.

## Stepwise Procedures

This protocol is presented in accordance with the 2013 SPIRIT (Standard Protocol Items: Recommendations for Interventional Trials) Statement, which was developed to provide guidance in the form of a checklist of recommended items to include in a clinical trial protocol to help improve the content and quality (25). This study was approved by Ethical Committee of National Center of Neurology and Psychiatry.

The schedule of enrollment, interventions, and assessments is summarized in Table 1. Participants will be recruited mainly by referrals from psychiatrists in National Center of Neurology and Psychiatry, Tokyo, Japan. We expect that two patients can be recruited per month on average, and that it will be possible to recruit 50 participants in 25 months.

All raters are well trained. All data will be administered in the EDC system, HOPE eACReSS, created by Fujitsu, Tokyo, Japan. Allocation and other identifiable data of subjects will be stored in a computer disconnected from Internet.

A review of previous studies indicates that most common adverse events included itching, tingling, headache, burning sensation, and discomfort (26). Experienced psychiatrists will check adverse effects before/immediately after every session, and evaluate the safety at every visit during the intervention. Data supervision using the EDC system will be conducted by an independent team of data managers and monitors.

## Anticipated Results

As discussed earlier, we anticipate that 9.00 will be the mean difference in the UPSA-B between two groups. Statistical analysis will be conducted using SAS 9.4, created by SAS Institute Inc., NC, USA. We will handle missing data with last observation carried forward method as an intention-to-treat analysis for all participants allocated. We will also perform a per protocol approach as a sensitivity analysis for the comparison of the results. For the UPSA-B, BACS, and PANSS, we will use analysis of covariance regarding each value at baseline as a covariate.

Functional capacity, or daily-living skills, has been reported to provide one of the most important factors affecting social consequences in patients with schizophrenia (6, 27). As stated before, tDCS is a safe method of brain stimulation and has been reported to improve several domains of cognition in schizophrenia (18–22). In addition, our pilot open study (22) demonstrated tDCS improved functional capacity, as measured by the UPSA-B. So far, no controlled trial has been performed to investigate the effect of tDCS on this level of functional outcome. Therefore, the randomized controlled trial described in this article is expected to provide a strategy to enhance social consequences in patients with schizophrenia.

The topic of adherence might be regarded as a potential pitfall in this protocol. However, as mentioned before, by adding costs of transportation for all included patients to the study budget, we plan to compensate for this issue. Also, the study coordinator will remind and reschedule all visits of participants as needed.

We believe that this is a well-designed controlled trial to test the ability of tDCS to improve an important determinant of outcome in patients with psychiatric disorders. Even if the results do not prove our hypothesis, the gathered data will contribute to a field that has not been widely studied.

## Ethics Statement

The protocol was presented to an institutional review board for approval (National Center of Neurology and Psychiatry Ethics Committee). The principal investigator (TS) will be responsible for conducting the informed consent process with all the study participants. All subjects must give consent to participate in the trial. Any relevant changes in the study protocol and/or the informed consent will be sent to the institutional review board as a protocol amendment. Identities of all subjects will be protected with an individual code. The protocol was registered in UMIN before starting the trial.

## Author Contributions

TS initiated the study. KM, TS, and ZN designed it and wrote the protocol. ZN and KS managed the literature searches and wrote the first draft of the manuscript. TI, TS, and ZN will administer tDCS. All the authors made substantial contribution, drafted the manuscript, and approved the final manuscript.

## Conflict of Interest Statement

The authors declare that the research was conducted in the absence of any commercial or financial relationships that could be construed as a potential conflict of interest.
